# Presence of Rheumatoid Factor during Chronic HCV Infection Is Associated with Expansion of Mature Activated Memory B-Cells that Are Hypo-Responsive to B-Cell Receptor Stimulation and Persist during the Early Stage of IFN Free Therapy

**DOI:** 10.1371/journal.pone.0144629

**Published:** 2015-12-09

**Authors:** Elane Reyes-Avilés, Lenche Kostadinova, Anne Rusterholtz, Angelica Cruz-Lebrón, Yngve Falck-Ytter, Donald D. Anthony

**Affiliations:** 1 Department of Pathology, Case Western Reserve University, Cleveland Ohio, United States of America; 2 Divisions of Infectious and Rheumatic Diseases, Department of Medicine, University Hospitals of Cleveland, Case Western Reserve University, Cleveland Ohio, United States of America; 3 Department of Medicine, VA Medical Center, Case Western Reserve University, Cleveland Ohio, United States of America; 4 Division of Gastroenterology, Veterans Administration Medical Center, Case Western Reserve University, Cleveland Ohio, United States of America; University of Montreal Hospital Research Center (CRCHUM), CANADA

## Abstract

Approximately half of those with chronic hepatitis C virus (HCV) infection have circulating rheumatoid factor (RF), and a portion of these individuals develop cryoglobulinemic vasculitis. B cell phenotype/function in relation to RF in serum has been unclear. We examined B cell subset distribution, activation state (CD86), cell cycle state (Ki67), and ex-vivo response to BCR, TLR9 and TLR7/8 stimulation, in chronic HCV-infected donors with or without RF, and uninfected donors. Mature-activated B-cells of HCV-infected donors had lower CD86 expression compared to uninfected donors, and in the presence of RF they also showed reduced CD86 expression in response to BCR and TLR9 stimulation. Additionally, mature activated memory B cells of HCV RF+ donors less commonly expressed Ki67^+^ than HCV RF- donors, and did not proliferate as well in response to BCR stimulation. Proportions of mature-activated B cells were enhanced, while naïve B-cells were lower in the peripheral blood of HCV-RF+ compared to RF- and uninfected donors. None of these parameters normalize by week 8 of IFN free direct acting antiviral (DAA) therapy in HCV RF+ donors, while in RF- donors, mature activated B cell proportions did normalize. These data indicate that while chronic HCV infection alone results in a lower state of activation in mature activated memory B cells, the presence of RF in serum is associated with a more pronounced state of unresponsiveness and an overrepresentation of these B cells in the blood. This phenotype persists at least during the early time window after removal of HCV from the host.

## Introduction

There are an estimated 170 million people worldwide chronically infected with Hepatitis C virus (HCV) [1] and 3.4–4.4 million in the US [2]. HCV targets the liver, where chronic infection can result in cirrhosis, liver failure and hepatocellular carcinoma [3, 4]. HCV infection also leads to autoimmunity, including cryoglobulinemia characterized by the accumulation of complexes composed of IgG bound to HCV and an IgM that binds to the Fc portion of IgG (rheumatoid factor or RF activity)[5–7]. Symptoms include weakness and arthralgia, and in more severe cases, neuropathy, renal disease and mortality can occur [8, 9]. The prevalence of cryoglobulins and RF ranges from 19%-50% during chronic HCV infection[10].

In healthy donors, naïve and resting memory B cells are the most common in the blood[11]. In chronic HIV infection, mature activated memory B cells (CD21^-/lo^ CD27^+^) show decreased proliferative capacity and enhanced CD86 expression [12, 13]. In HCV infection with cryoglobulinemia, CD21^lo^ CD27^+^ B cells have decreased mobilization of calcium after BCR stimulation [14, 15]. Decreased BCR signaling has also been observed in CD21^+^ CD27^+^ B cells of HCV donors with cryoglobulinemia[16]. Further characterization is required to asses which B cell subset (CD21^-/lo^CD27^+^ or CD21^+^ CD27^+^) is dysregulated during HCV infection. We have shown that in HCV infection there are increased proportions of mature activated B cells, and this is associated with lower cell cycling (ki-67 expression)[17]. Whether these observations are linked to RF status was unknown. Our results here indicate that RF positivity is the dominant factor associated with enhanced proportions of mature activated memory B cells and these cells have a phenotype associated with a state of unresponsiveness. In spite of over 4 weeks of viral clearance, this overrepresentation of hypo-responsive mature activated memory B cells remains in HCV RF+ donors but not HCV RF- donors.

## Materials and Methods

### Study participants

Chronic HCV infected subjects RF- (n = 23), RF+ (n = 20) and uninfected donors (n = 23) were enrolled from the Cleveland VA and University Hospitals for venous blood sampling under Cleveland VA Medical Center and University Hospitals of Cleveland approved Institutional Review Board protocols. All patients provided written informed consent in accordance with the Declaration of Helsinki. HCV infected subjects were serum HCV antibody positive for at least 6 months, HCV RNA positive by PCR and untreated for HCV infection. RF status was determined by ELISA (below). Uninfected donors were HCV and HIV antibody negative, and were derived from the Cleveland VA patient population attending general medical clinic. Characteristics of the study subjects are shown in **Table 1**. Age, race/ethnicity, and sex did not differ. Out of the 20 HCV RF+ donors, 5 were screened for cryoglobulins in serum and were negative.

**Table 1:** Untreated study donor characteristics.

Variable	Uninfected donors	HCV Rheumatoid Factor negative	HCV Rheumatoid Factor positive
**Number**	N = 23	N = 23	N = 20
**Age, years**	59 (42–83)	62 (28–72)	59 (50–64)
**Plasma HCV RNA level, IU/mL**		508,848 (69,124–3,507,690)	1,462,925 (316,393–6,530,280)^b^
**Serum Albumin level, g/dL**	4.1 (3.5–4.9)	3.8 (3.4–4.5)^a^	3.5 (2.4–4.3)^a^
**Platelet count, x10** ^**3**^ **/uL**	232 (160–345)	195 (123–400)	203 (81–323)
**Serum AST level, U/L**	19 (13–40)	38 (24–199)^a^	48 (26–282)^a^
**Serum ALT level, U/L**	20 (11–65)	46(17–316)^a^	51.5 (31–235)^a^
**APRI**	0.209 (0.084–0.520)	0.448 (0.148–2.44)^a^	0.619 (0.187–6.19)^a^
<0.4	22 (95.6%)	10 (43.5%)	6 (30%)
0.4–1.5	1 (4.4%)	10 (43.5%)	9 (45%)
>1.5		3 (13%)	5 (25%)
**HCV genotype**			
1		21 (91.3%)	18 (90%)
2/3		2 (8.7%)	2 (10%)
**Duration of HCV infection**			
1–10 years		2 (8.7%)	1 (5%)
>10 years		20 (86.9%)	16 (69.6%)
Unknown		1 (4.4)	3 (25.4%)
**Sex**			
Male	23 (100%)	22 (95.6%)	20 (100%)
Female	0	1 (4.4%)	
**Ethnicity**			
Caucasian	6 (26.1%)	9 (39.1%)	5 (25.0%)
African American	16 (69.6%)	14 (60.9%)	14 (70.0%)
Hispanic White			1(5%)
Native Hawaiian	1 (4.3%)		
**Absolute CD19+ count (cells/uL)**	219.8 (128.7–940.2)	243.55 (73.0–986)	210.6 (109–820.0)
**RF level IU/mL**			80% RF<100 IU/mL 20% RF >100 IU/mL

Values are expressed as median (range) for age, HCV RNA level, albumin, platelet, AST level, ALT level, APRI score, calculated as described previously [47], and absolute CD19+ count. Numbers and proportions of subjects within each category are given for HCV genotype, duration of HCV infection, sex, ethnicity and RF. Abbreviations: ALT, alanine aminotransferase; APRI, AST-to-Platelet ratio index; AST, aspartate aminotransferase; HCV, hepatitis C virus, and RF, rheumatoid factor. ^a^ p< .05 compared with uninfected donors; ^b^ p< .05 compared between HCV RF- and HCV RF+

### Longitudinal IFN free direct acting antiviral (DAA) therapy samples

Peripheral blood samples from 20 patients receiving IFN free DAA therapy (Sofosbuvir 90 mg/Ledipasvir 400mg/ weight-based ribavirin (1000–1200 mg/day therapy n = 19, or Ombitasvir, Paritaprevir Co-dosed With Ritonavir, Dasabuvir, Ribavirin, n = 1) were obtained at baseline and at week 8. Entry criteria included chronic HCV infection (>6 months seropositive or RNA positive), genotype 1 HCV, and absence of serum Hepatitis B surface antigen and HIV antibody. Baseline characteristics of the 20 study subjects are shown in **S1 Table**. None were screened for cryoglobulins in serum.

### Flow Cytometric Analysis

Nine parameter flow cytometric analysis was performed on 200uL of whole blood. Lymphocytes were assessed by forward and side scatter, and stained for the following: anti-CD19-PECy5 (clone HIB19),anti- CD20- APC-H7 (clone L27), anti-CD10- APC (clone HI10a), anti-CD21- PE (clone B-ly4), anti- CD27- PECy7 (clone M-T271) (all from BD Biosciences, San Jose, CA) and anti-CD38- Alexa Fluor 700 (clone HIT2, Biolegend, San Diego, CA). At least 20,000 CD19+ B cells events were acquired on an LSRII cytometer driven by FACSDiVa version 6.2 software. Analysis was performed using FlowJo Version 10 software. BD TruCOUNT (BD Biosciences) beads were used for absolute counts of CD19+ B cells. For CD86 FITC (clone 2331, BD Biosciences, San Jose, CA) whole blood was stained for 10 minutes in the dark at room temperature, then lysed for 2 minutes with 2mL of BD Lyse (BD Pharmigen, San Diego, CA) and washed with 2mL of 0.1% BSA+ PBS, then fixed with 200uL of BD Stabilizing Fixative (BD Biosciences). For Ki-67(clone B56, BD Biosciences),whole blood was stained as above, but permeabilized with BD Cytofix/Permeabilization (BD Biosciences) on ice for 20 minutes, washed with 2mL of Perm Wash (BD Biosciences) and stained with 20uL of anti-Ki-67 FITC for 30 minutes on ice, washed twice with 2 mL of Perm wash and fixed.

### Mature activated memory B cell sort and Phosflow assay

B cells were enriched by Rosette Sep (Stemcell, Vancouver, Canada) from 100ml blood of uninfected (n = 3) and HCV RF+ (n = 3) donors. Mature activated memory B cells (CD19+CD20+CD21-/lo+CD27+) were sorted using a FACS Aria and 3,000 cells were stimulated for 5 minutes with RPMI 1640 alone or RPMI 1640 with anti-IgG BCR (10ug/mL anti-Fab2 IgG fragment, Jackson ImmunoResearch West Grove, PA). Cells were fixed with 1ml of BD Cytofix (BD Biosciences) and incubated at 37°C for 10 minutes. Cells were washed with 2 mL PBS, then suspended in 500uL of cold 90% methanol+ PBS and stored at -20°C for 30 minutes. Samples were then washed with 2 mL of PBS and 2mL of PBS+ 0.1%BSA, then incubated with 20 uL of anti-Syk (pY348) (clone I120-722, Biolegend San Diego, CA) antibody for 60 minutes on ice. Cells were washed with PBS+ 0.1%BSA, fixed, and acquired as above.

### IgM Rheumatoid Factor (RF) detection

Serum samples were collected using Vacutainer serum tubes (BD Biosciences) and frozen at -20°C. IgM RF was measured by ELISA (ALPCO, Salem, NH). Detection limit is 25 IU/mL, samples greater than 25 IU/mL of IgM RF were considered positive.

### BCR, TLR9 and TLR7/8 induced CD86 upregulation by flow cytometry

Peripheral blood mononuclear cells (PBMC) were isolated using Ficoll (Fisher Scientific, Hudson, NH) and centrifugation. Where indicated, 5x10^6^ PBMCs were cultured for 3 days in the presence of anti-BCR (4ug/mL anti IgG Fab’2 fragment, Jackson Immunoresearch Laboratories, West Grove, PA), TLR9 agonist (CpG 2006;1ug/mL, Operon, Huntsville, AL) or TLR7/8 agonist (R848;1ug/mL, Invivogen, San Diego, CA). At day 3, PBMC were stained for B cell subset markers, yellow viability dye (Invitrogen, Carlsbad, CA) and CD86 FITC (clone 2331, BD Biosciences, San Jose, CA). Flow cytometric acquisition was performed on 30,000–50,000 CD19+ B cells as previously mentioned.

### Proliferation by analysis of CFSE dye dilution

PBMCs (3x10^6^-5x10^6^) were labeled with 1.2 uM of 5(6)-carboxyfluorescein diacetate, succinimidyl ester (Molecular Probes Invitrogen, Grand Island, NY) in the presence of 0.2% BSA+ PBS [18]. To quench the staining, 2.5 mL of FBS was added and incubated on ice for 5 minutes. Cells were washed with 0.2% BSA and cultured in complete RPMI 1640 (1%penicillin/streptomycin and 1% L-glut with 10% FCS), anti BCR (4ug/mL anti-Fab_2_ IgG fragment, Jackson ImmunoResearch) or CpG 2006 (1ug/mL, Operon Huntsville, AL), and the combination of BCR and CpG 2006. Cells were stained at day 6 for B cell subset markers, and 30,000–50,000 CD19+ events were acquired as previously mentioned.

### Statistical Analysis

Non-parametric Mann- Whitney U tests were performed to determine significance of differences between two groups. A non-parametric Kruskal- Wallis test was used to evaluate differences among more than two groups. We performed a paired *t* test to compare longitudinally collected samples from IFN free treated donors. Analyses were performed using Graph Pad Prism Version software 6. Values p ≤ 0.05 were considered statistically significant.

## Results

### Mature activated memory B cells of HCV donors less commonly express CD86 and have limited response to BCR stimulation when serum RF is present

Previous studies have characterized B cell phenotype and function in the setting of cryoglobulinemia, but the assessment of cryoglobulins in the serum varies widely both in clinical practice and in the published literature [19]. RF is a constituent of cryoglobulins [20] and its detection is reproducible. We focused on the presence of RF in HCV infection and its association with B cell phenotype and function.

**Fig 1:**
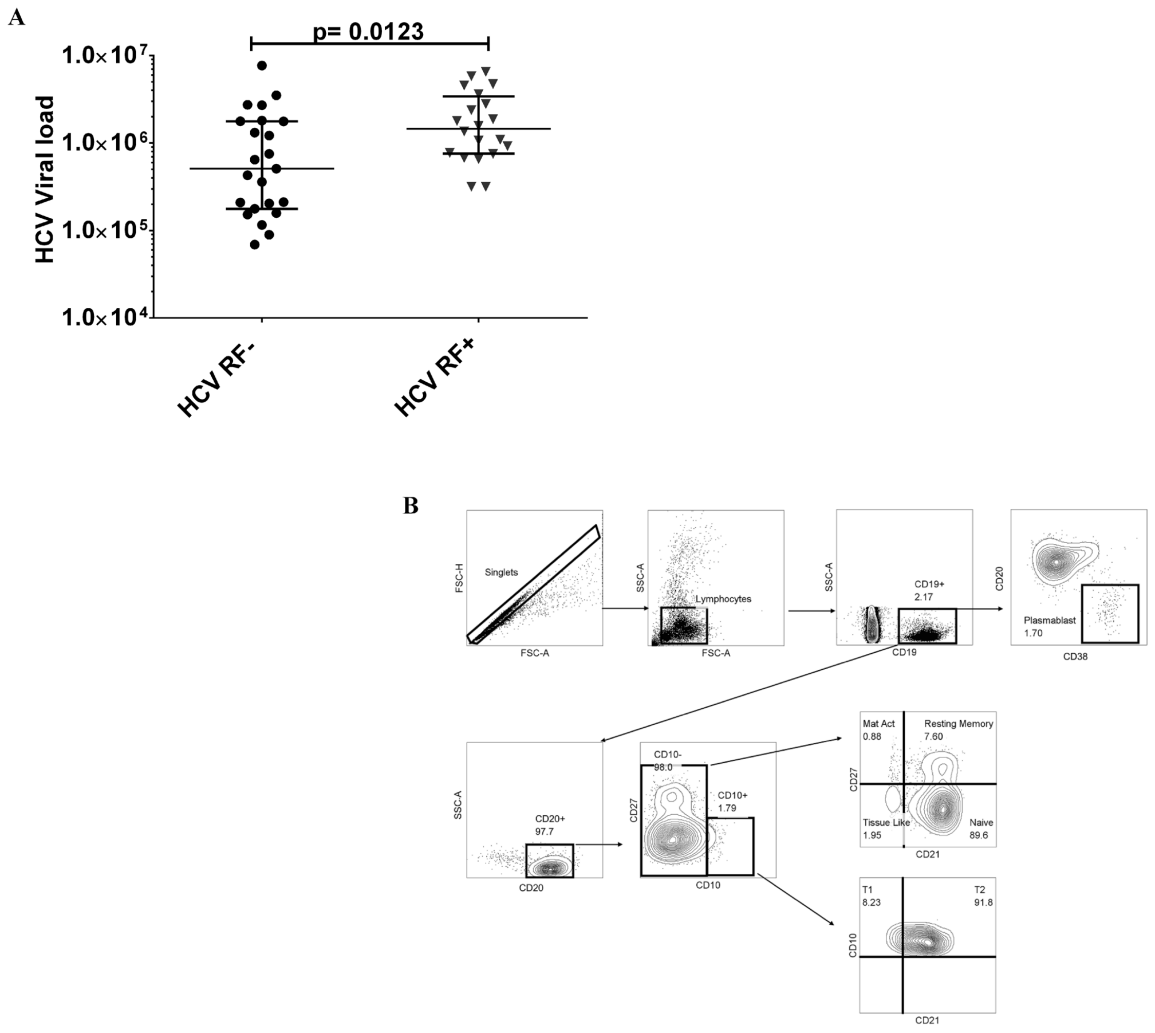
Increased viral load in HCV RF+ donors and gating strategy to enumerate B cell subset frequencies from whole blood. **(**A), HCV RNA copies IU/mL HCV RF- (●, n = 23) and HCV RF+ (▼, n = 23). **(**B) Shown an uninfected donor sample for total immature transitional B cells (CD19+CD20+CD10+) and its differentiation stages: T1 stage (CD19^+^CD20^+^CD10^+^CD21-) and T2 stage (CD19^+^CD20^+^CD10^+^CD21^+^). Mature activated B cells(CD19^+^CD20^+^CD10^-^CD21^-^CD27^+^), resting memory B cells (CD19^+^CD20^+^CD21^+^CD27^+^), naïve (CD19^+^CD20^+^CD21^+^CD27^-^),Tissue like memory B cells (CD19^+^CD20^+^CD10^-^CD21^-^CD27^-^) and Plasmablast cells (CD19^+^CD20^-^CD38^+^)

First, plasma HCV RNA levels were higher in HCV RF+ donors when compared to HCV RF- donors (p = 0.0123) (**Fig 1A)**. Hence, B cells of HCV RF+ donors might reflect an increased state of activation as a result of greater antigen and virus stimulation. We characterized B cells as follows (**Fig 1B)**: Immature transitional (CD19^+^CD20^+^CD10^+^) further characterized as T1 stage Immature Transitional (CD19^+^CD20^+^CD10^+^CD21^-^) and T2 stage Immature Transitional (CD19^+^CD20^+^CD10^+^CD21^+^) [21], Naïve (CD19^+^CD20^+^ CD10^-^CD27^-^ CD21^+^), Mature activated memory (CD19^+^CD20^+^CD10^-^CD27^+^CD21^-^), Resting memory (CD19^+^CD20^+^ CD10^-^CD27^+^CD21^+^), Tissue like memory (CD19^+^CD20^+^ CD10^-^CD27^-^CD21^-^) and Plasmablast cells (CD19^+^CD20^-^CD38^+^).

**Fig 2:**
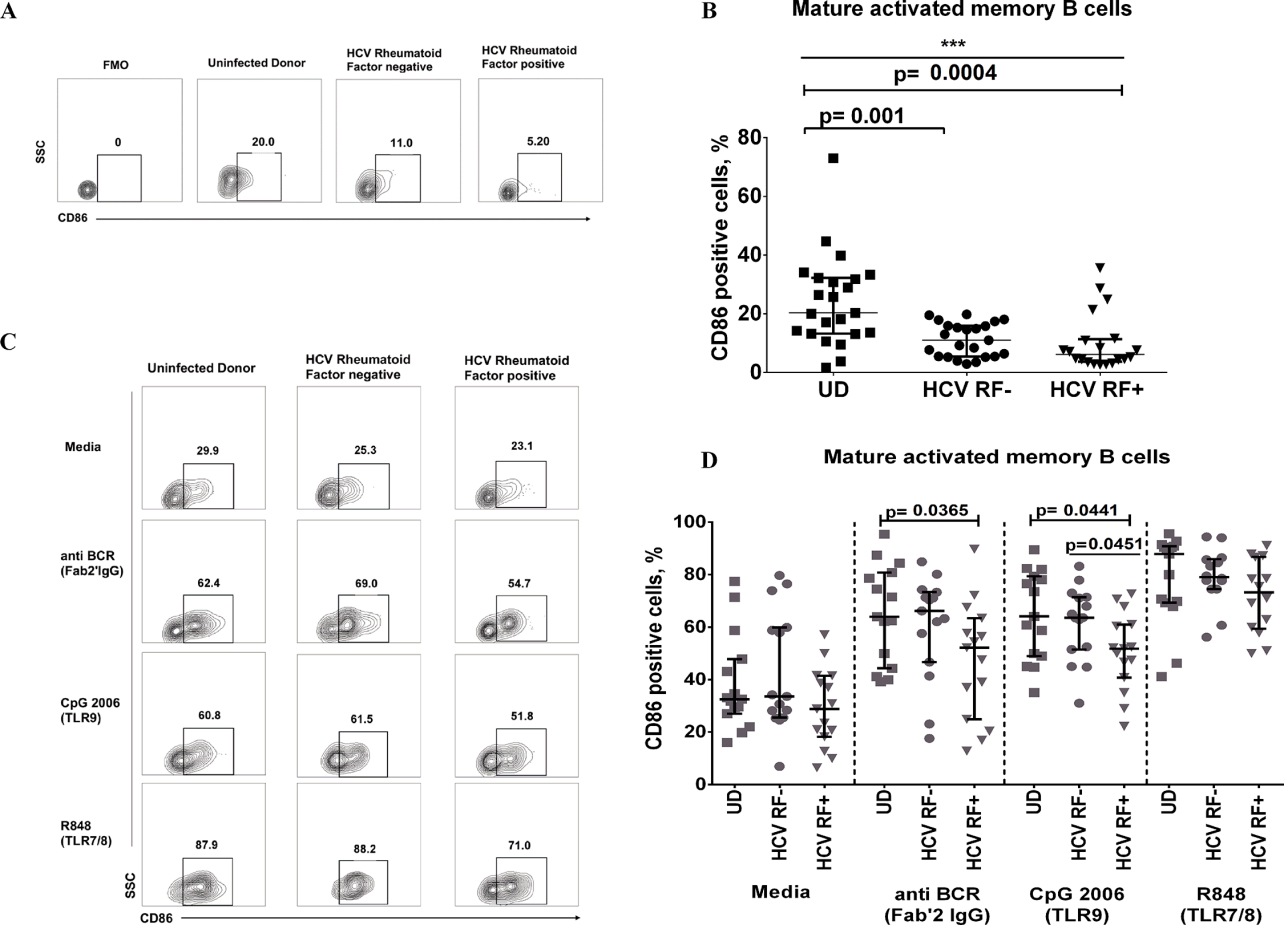
Mature activated memory B cells of HCV donors show low CD86 expression and have limited response to BCR stimulation in HCV RF+ donors. **(**A-B) Representative and summary for CD86 expression in whole blood of uninfected donors (UD **▄** n = 23), HCV Rheumatoid Factor negative (HCV RF-, **●** n = 23) and HCV Rheumatoid Factor positive (HCV RF+, **▼** n = 20). (C-D) Representative and summary data of CD86 ex vivo experiments, where peripheral blood mononuclear cells were cultured for 3 days in medium alone, anti IgG F(ab’)^2^ fragment (4ug/mL), CpG (1ug/mL), R848 (Iug/mL) and cells were stained for B cell subset markers and CD86. UD(▄, n = 15), HCV RF- (●, n = 14), HCV RF+ (▼, n = 15). Kruskal- Wallis was used to analyzed differences among the three groups (*) of donors and Mann-Whitney U test (p≤0.05) was used between two groups of donors.

CD86 (B7.2) is known to be up-regulated in states of B cell activation and facilitates T cell-B cell interaction [22]. We identified that only circulating mature activated memory B cells (CD19^+^CD21^-^/^lo^CD27^+^) from HCV donors have reduced CD86 expression compared to uninfected donors (UD), irrespective of the presence of RF (HCV RF-:11.0% vs UD: 24.30%, p = 0.001; HCV RF+: 6.14% vs UD: 24.30%, p = 0.003) **(Fig 2A and 2B).** Plasmablast B cells have the highest expression of CD86 in our cohorts **(S1A Fig**). B cells express TLR7 and TLR9 [23], and its stimulation increases CD86 expression [24, 25]. Maximal expression of CD86 occurs at 72 hours with these stimuli [26]. We assessed if mature activated memory B cells can increase CD86 expression after 3 day stimulation with BCR (F (ab’)_2_ IgG), TLR9 (CpG2006) or TLR7/8 (R848). Surprisingly, only the mature activated memory B cells of HCV RF+ donors showed markedly lower CD86 expression after BCR stimulation (HCV RF+: 52.2% vs UD 62.4%, p = 0.0365) and CpG 2006 stimulation (HCV RF+: 51.8% vs HCV RF- 63.5%, p = 0.0441; HCV RF+ 51.8% vs UD 64.1%, p = 0.0451) **(Fig 2C and 2D)**. To confirm whether anti BCR stimulation (F(ab’)_2_ IgG fragment) was directly active on mature activated B cells, we flow sorted mature activated memory B cells of 3 HCV RF+ and 3 uninfected donors and performed a phosflow for phosphorylated Syk Y348 [27]. Indeed, F (ab’)_2_ IgG stimulation leads to increased pSyk Y348 after 5 minutes of stimulation in both HCV RF+ and UD **(S1B and S1C Fig)**. Overall, CD86 expression is lower on mature activated memory B cells of HCV RF+ and HCV RF- donors in the peripheral blood, but only HCV RF+ mature activated memory B cells are hypo-responsive to further stimulation *ex vivo*.

### Mature activated memory B cells of HCV RF+ donors show decreased Ki67 expression and less commonly proliferate in response to BCR stimulation

**Fig 3:**
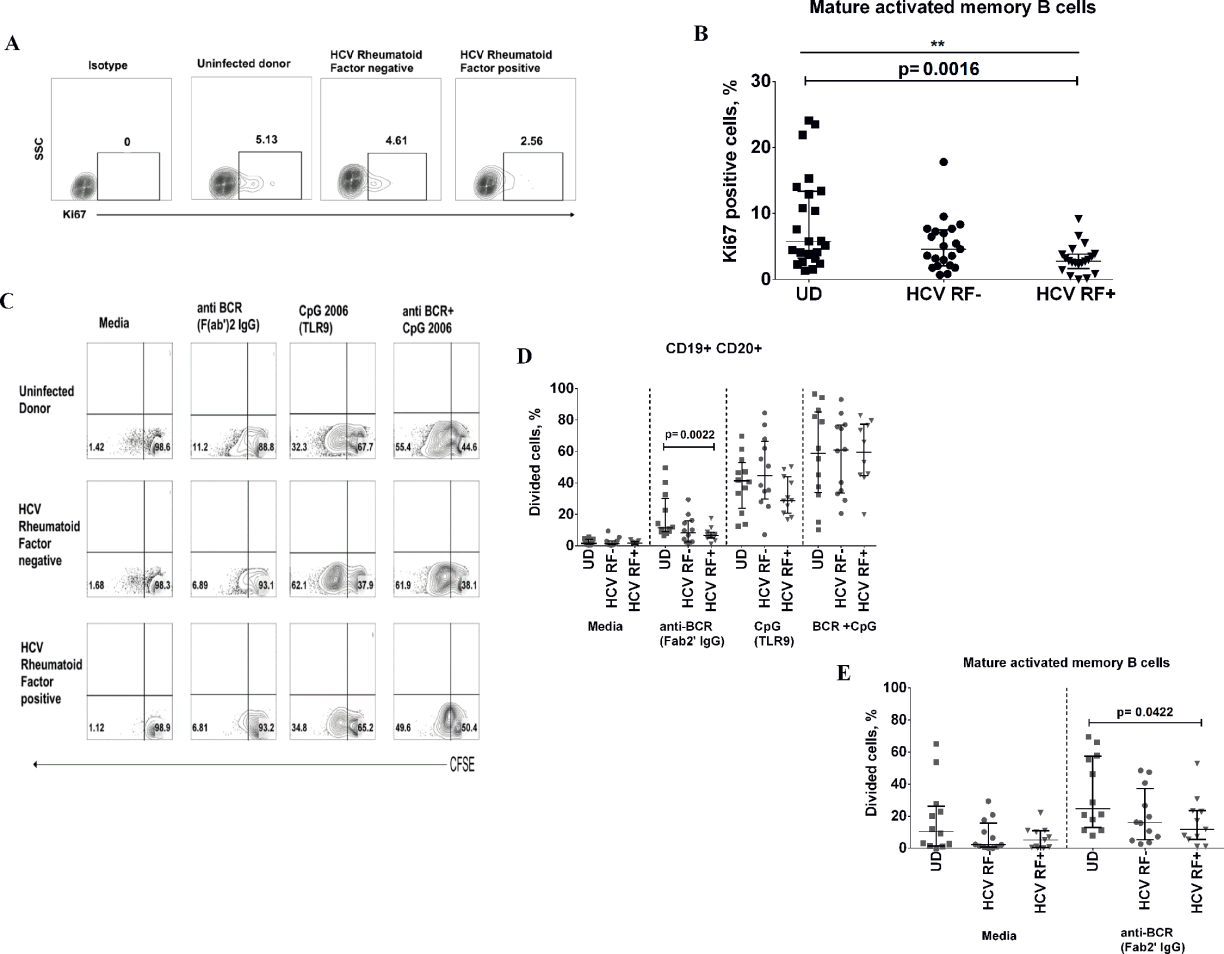
Mature activated memory B cells of HCV RF+ donors have decreased ki-67 and are less likely to proliferate in response to BCR stimulation. **(**A-B) Representative and summary for Ki-67expression in whole blood of uninfected donors (UD **▄** n = 23), HCV RF- (**●** n = 20) and HCV RF+ (**▼** n = 20). (C-D) Representative and summary data of CFSE dye dilution of ex vivo experiments, where peripheral blood mononuclear cells were cultured for 6 days in medium alone, anti BCR (anti IgG F(ab’)^2^ fragment, 4ug/mL), CpG 2006 (1ug/mL), R848 (Iug/mL) and cells were stained for B cell subset markers. UD(▄, n = 11), HCV RF- (●, n = 11), HCV RF+ (▼, n = 10). Kruskal- Wallis was used to analyzed differences among the three groups (*) of donors and Mann-Whitney U test (p≤0.05) was used between two groups of donors.

HCV directly promotes proliferation of naïve (CD27^-^) and memory (CD27^+^) B cells via its binding to CD81 [28]. We next investigated the cell cycle state (Ki67 expression) of B cell subsets in peripheral blood. Mature activated memory B cells of HCV RF+ donors have significantly lower Ki67 expression than uninfected donors (HCV RF +: 2.76% vs UD: 5.77%, p = 0.0016), whereas no difference was observed between HCV RF+ and HCV RF- donors **(Fig 3A and 3B)**. This was not attributable to differences in bulk B cell (CD19^+^ CD20^+^) Ki67 expression, and we did not observe differences in cell cycle state in other B cell subsets (data not shown). Plasmablast had the highest expression of Ki67 **(S2A Fig)**.Next, we evaluated ex-vivo proliferative potential by CFSE dye dilution assay of PBMCs isolated from untreated HCV infected donors with or without RF and uninfected donors. Cells were cultured for 6 days in the presence of anti BCR (F(ab’)_2_ IgG fragment), TLR9 (CpG 2006) or the combination of stimuli. CpG 2006 has been shown to induce proliferation of memory and naïve B cells in the absence of T cell help [29, 30]. Bulk B cells (CD19^+^CD20^+^) of HCV RF+ donors are less proliferative than those of uninfected donors in the presence of BCR stimulation (HCV RF+: 6.21% vs UD: 11.45%, p = 0.002) **(Fig 3C and 3D)**. HCV RF+ donors bulk B cells also showed a trend towards decreased responsiveness to TLR9 agonist in comparison to HCV RF- donors(p = 0.09). Mature activated memory B cell showed a similar pattern as observed in bulk B cells where they are less proliferative in the presence of BCR stimulation (HCV RF+:11.8% vs UD: 24.8%, p = 0.042) **(Fig 3E)**. In summary, circulating mature activated memory B cells of HCV RF+ donors are less commonly in cell cycle compared to uninfected donors and these B cells have a reduced proliferative response to BCR stimulation when RF is present.

### Rheumatoid factor in HCV donors is associated with enhanced proportions of mature activated memory B cells and reduced proportions of naïve B cells in whole blood

**Fig 4:**
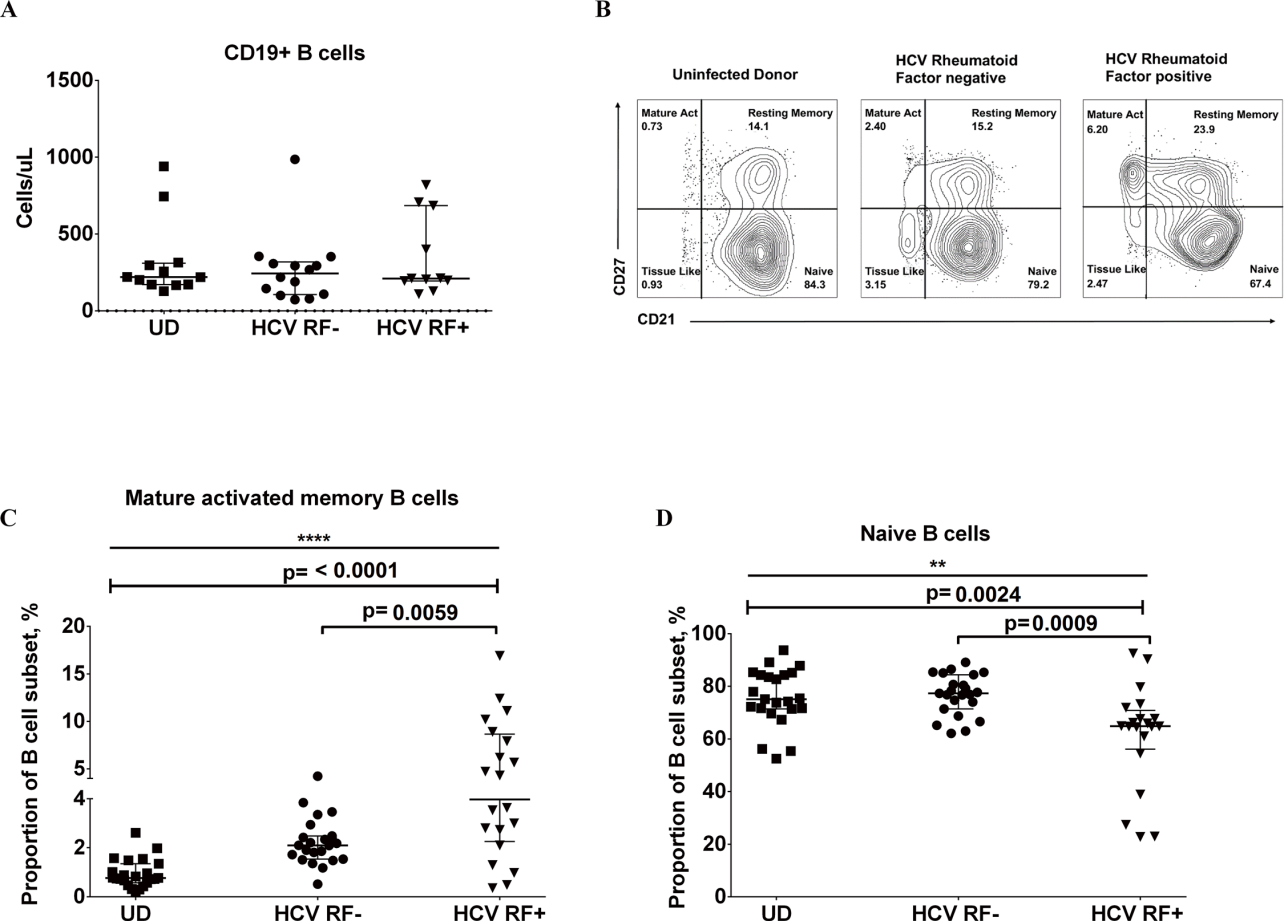
HCV RF+ (donors have greater proportions of mature activated memory B cells and reduced proportions of naïve B cells in comparison to uninfected donors and HCV RF- donors. **(**A) Absolute count of CD19+ B cell per uL of whole blood. (B) Representative flow plot of altered B cell subset distribution in whole blood from Uninfected donor, HCV RF-, and HCV RF+ donors. (C) Summary data of Proportion of mature activated memory B cells (%) in whole blood (CD19+CD20+CD10-CD21-/loCD27+) for Uninfected donors (**▄ n = 23)**, HCV RF- **(● n = 23)**, and HCV RF+ donors (**▼, n = 20).** (D). Summary data of Proportion of naïve B cells (CD19+CD20+CD10-CD21+CD27-) in whole blood for Uninfected donors (**▄ n = 23)**, HCV RF- **(● n = 23)**, and HCV RF+ donors (**▼, n = 20)**. Kruskal- Wallis was used to analyzed differences among the three groups of donors and Mann-Whitney U test was used between two groups of donors (p≤0.05)

Circulating mature activated memory B cells from untreated HCV infected donors show decreased activation and proliferation, but only HCV RF+ donors show limited activation to *ex vivo* stimulation. We next examined if B cell subset distribution is altered in HCV RF+ donors. Absolute counts of CD19+ B cells are similar among uninfected, HCV RF- and HCV RF+ donors **(Fig 4A).** Mature activated B cell proportions were greater in HCV RF+ donors when compared to uninfected donors (HCV RF+: 3.9% vs UD: 0.77%, p = 0.0002) and to HCV RF- donors (HCV RF+: 3.9% vs HCV RF-: 2.1%, p = 0.008) (**Fig 4B and 4C**). Naive B cell proportion was significantly lower in HCV RF+ donors compared to uninfected donors (HCV RF+: 64.8% vs UD: 75.1%, p = 0.0024) and to HCV RF- donors (HCV RF+: 64.8% vs HCV RF-: 77.3%, p = 0.0009) (**Fig 4D**). We found no evidence that resistance to cell death is involved in this overrepresentation of mature activated memory B cells or reduction of naïve B cells in HCV RF+ donors by comparison of Bcl-2 expression and Annexin V on these subsets among groups (data not shown). Taken together, the presence of RF in chronic HCV infection is associated with greater proportions of mature activated memory B cells whereas naïve B cell proportions are lower.

### Short term viral eradication does not lead to complete removal of RF from serum or restoration of B cell subsets distribution in HCV RF+ donors

**Fig 5:**
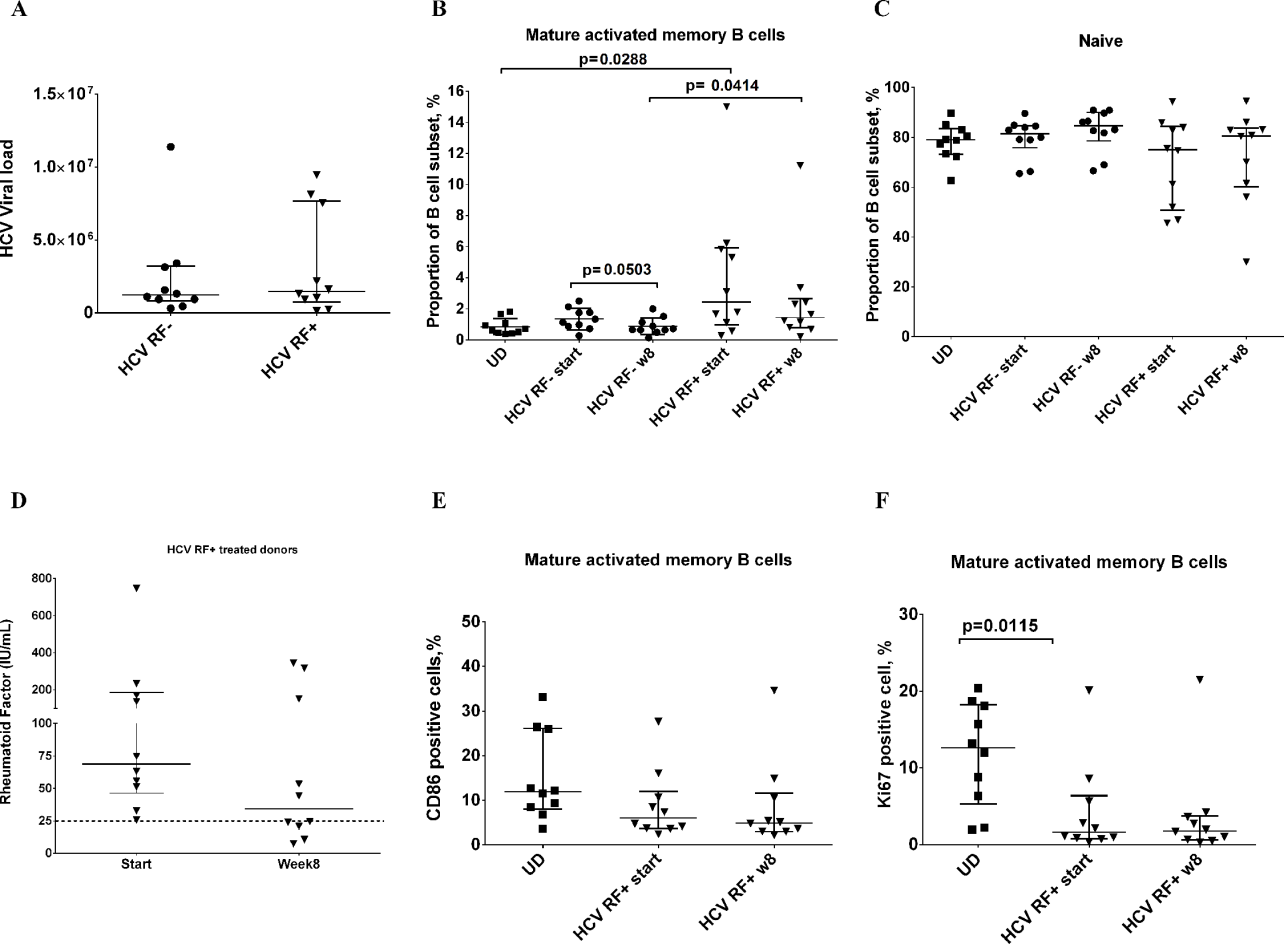
HCV treatment does not lead to removal of Rheumatoid Factor from serum or B cell restoration. **(**A) Plasma HCV RNA level, IU/ml in HCV RF- donors **(● n = 10)** and HCV RF+ donors (**▼, n = 20)**. (B) Summary data of proportion of mature activated memory B cells (%) in frozen PBMCs (CD19+CD20+CD10-CD21-/loCD27+) for Uninfected donors (**▄ n = 10)**, HCV RF- **(● n = 10)**, and HCV RF+ donors (**▼, n = 10). (**C) Summary data of proportion of naïve B cells (CD19+CD20+CD10-CD21+CD27-) in frozen PBMCs for Uninfected donors (**▄ n = 10)**, HCV RF- **(● n = 10)**, and HCV RF+ donors (**▼, n = 10)**.(D) Summary data of CD86 expression on mature activated memory B cells (CD19+CD20+CD10-CD21-/loCD27+) for Uninfected donors (▄ n = 10) and HCV RF+ donors (▼, n = 10). (E) D) Summary data of Ki67 expression on mature activated memory B cells (CD19+CD20+CD10-CD21-/loCD27+) for Uninfected donors (▄ n = 10) and HCV RF+ donors (▼, n = 10). Kruskal- Wallis was used to analyzed differences among the three groups of donors (*) and Mann-Whitney U test was used between two groups of donors (p≤0.05)

Clearance of HCV virus leads to a decrease in cryoglobulins (composed of RF) within 24 months [31]. Therefore, viral clearance should eventually eliminate RF from the serum. New combination therapies such as sofosbuvir/ledipasvir have improved viral clearance to undetectable levels as early as 1–4 weeks [32]. At the start of therapy the HCV infected RF+ and RF- donors had comparable viral loads (**Fig 5A**), and at 8 weeks on therapy 100% of subjects had undetectable viral levels (less than15IU/mL) (**S1 Table**). After 8 weeks of treatment, proportions of mature activated memory B cells of HCV RF+ donors showed a slight decrease when compared to the baseline levels but this was not statistically significant, or comparable to levels of uninfected donors (HCV RF+ week 8: 1.44% vs UD: 0.80%) **(Fig 5B)**. In contrast, proportions of mature activated memory B cells of HCV RF- donors declined after 8 weeks of treatment (HCV RF- baseline: 1.34% vs. week 8: 0.88% p = 0.050) and to similar levels as uninfected donors (0.80%) **(Fig 5B)**. Naïve B cell proportions of HCV RF+ donors showed a modest trend towards an increase at week 8 of treatment (baseline:75.1% vs. week 8: 80.0%, p = 0.66) **(Fig 5C)**, with week 8 levels comparable to those of uninfected donors (UD: 79%). We observed that serum RF trends lower after 8 weeks of treatment in comparison to baseline (median baseline: 68.08 IU/mL vs. week 8: 34.3 IU/mL, p = 0.23), with 3 out of 10 donors (30%) negative for RF (less than 25 IU/mL) by week 8 **(Fig 5D).** B cell activation in mature activated memory B cells was also analyzed in this cohort of HCV RF+ treated donors. In spite of viral clearance, we observed that CD86 expression remained consistently low at week 8 in mature activated memory B cells of HCV RF+ donors (baseline: 6% vs week 8: 4.8%)**(Fig 5E)**. Ki67 expression was lower on mature activated memory B cells in HCV RF+ donors before treatment compared to uninfected donors (HCV RF+ 1.61% vs UD: 12.6%, p = 0.012) **(Fig 5F)**. Interestingly, Ki67 levels of HCV RF+ donors also remained low after 8 weeks of treatment (baseline: 1.61% vs week 8: 1.79%) **(Fig 5F)**. These data suggest that there is a delay in the restoration of B cell subset distribution and phenotype in HCV donors that have RF in their serum after 8 weeks of treatment with DAA HCV therapy, a time point where virus is no longer present.

## Discussion

We have shown that the presence of rheumatoid factor (RF) in HCV chronically infected donors is associated with decreased activation and proliferation within the mature activated memory B cell subset (CD21^-/lo^CD27^+^). This same subset is overrepresented within the peripheral blood. Persistence of RF after removal of HCV is accompanied by a delay in the normalization of activation, cell cycle and distribution of mature activated memory B cells in HCV RF+ donors, but no delay in normalization of the mature activated subset frequency of HCV RF- donors. Therefore, the presence of RF is tightly associated with B cell dysfunction and alteration in B cell subset proportions.

In HCV infected patients with cryoglobulinemia, marginal zone like B cells that are IgM+ CD21^lo^ CD27^+^ are enhanced in the periphery [14]. This B cell subset is low in CD21 expression, differentiating it from typical circulating marginal zone B cells (IgM+CD27+CD21^hi^) that are derived from the B2 lineage [14, 33, 34]. The marginal zone like IgM+ CD21^lo^ CD27^+^ B cells in HCV infected patients with cryoglobulinemia show reduced calcium flux in response to BCR stimulation [15]. Mature activated memory B cells (CD27+CD21^lo^) described here are similar to these marginal zone like B cells previously described. In particular we also find here a reduced responsiveness to BCR stimulation **(Fig 2D and Fig 3D)**. In contrast, while CD5 is described to be a marker of B1a B cells that have been characterized to produce RF in rheumatoid arthritis patients [35], RF expressing B cells in HCV chronic infection are CD5-[14]. Overall, mature activated B cells studied here are similar to marginal zone like B cells, though further functional characterization is warranted.

While the work of Ni et al observed that circulating CD19^+^ B cells in HCV donors had no increase in CD86 expression in comparison to healthy controls [36], findings of others indicated circulating memory B cells (CD19^+^CD27^+^) of HCV infected donors with cryoglobulinemia [37] and without cryoglobulinemia [38] have increased CD86 expression. Terrier et al found also that CD86 expression is increased at the RNA and protein level, and Ki67 expression is increased at the protein level in the CD21^lo^ CD27^+^ B cells of HCV infected cryoglobulinemic subjects [39]. In marked contrast to these previous reports, we found circulating mature activated memory B cells (CD27^+^CD21^-^/^lo^) of HCV infected donors to have decreased CD86 and Ki67 expression. Our study differs from these other studies by a lack of association between liver damage index (APRI) and CD86 or Ki67 expression compared to that of Santer et al, where memory B cells of HCV donors with advanced liver fibrosis had the highest level of CD86 expression [37]. Additionally, our study included only untreated HCV infected subjects, differing from studies by others [39]. Further features of our study that differ from these other studies include: real time analysis of whole blood samples, a male dominant population, uninfected donor controls that are closely matched in age and clinical characteristics and similar racial and socioeconomic factors.

To explore if mature activated memory B cells of HCV infected donors are responsive to further stimulation we isolated PBMC of HCV infected participants and found that this subset is unable to increase CD86 expression in response to BCR and TLR9 stimulus when RF is present in the serum (HCV RF+ donors). This is in support of the concept that the same functional state *in vivo* is observed in direct *ex vivo* assays. Also, in agreement with our data, Terrier et al showed that CD21^lo^ CD27^+^ B cells fail to upregulate activation markers (CD69, CD25) and proliferate after BCR stimulation [39]. In the mouse model of lupus (AM14 B cells), the BCR has RF activity and its stimulation fails to promote B cell activation (anergy) but TLR9 engagement leads to B cell activation and proliferation[40]. Hence, TLR9 can play a role in activating B cells in the presence of autoantigen [41]. Here, we observed that the *ex vivo* response to both BCR alone and TLR9 is limited in HCV RF+ donors in comparison to HCV RF- and UD, suggesting that the presence of RF is associated with a pronounced unresponsiveness to further stimulation.

We also sought to determine if mature activated memory B cells of HCV RF+ donors are responsive to BCR stimulation via phosflow assay of proximal signaling events. We observed that sorted mature activated memory B cells of HCV RF+ donors are in fact responsive to IgG stimulation since they can increase pSYK levels to 40% after stimulation, a magnitude comparable to uninfected donors (**S1B Fig**). Hence, proximal BCR signaling events may in fact be intact in these HCV RF^+^ mature activated cells, indicating a distal signaling event potentially impaired or inhibited. Perhaps productive BCR signaling is dampened by inhibitory complexes such as SHIP1 (SRC-homology-2-domain-containing inositol-5-phosphatase 1) and DOK (docking protein) [42]. Alternatively, another cell type within the PBMC fraction may be operating in a negative accessory cell regulatory fashion.

Overall, our data suggest that overrepresentation of circulating mature activated memory B cells of HCV RF+ donors is consistent with a profile of B cells that avoid deletion and persist in a state of unresponsiveness towards antigenic stimulation[43]. Enhanced proportions of mature activated memory B cells in whole blood of HCV donors has been previously described by others [44] and us [17]. We are the first to highlight that this overrepresentation is associated with the presence of RF during HCV infection, and not exclusive to cryoblobulinemia. In addition, we observed naïve B cell proportions (CD21^+^ CD27^-^) are reduced in HCV RF+ donors. We looked at CD21 expression (MFI) on naïve B cells to understand if downregulation of this marker is associated with the overrepresentation of mature activated memory B cells in HCV RF+ donors. Naive B cells of HCV RF+ donors showed reduced CD21 expression in marked contrast to HCV RF- donors (MFI: HCV RF+: 5569 vs MFI HCV RF-:7111, p = 0.0489) (**S2B Fig**). This finding is similar to a previous report in patients with rheumatoid arthritis and common variable immunodeficiency, where the naïve B cells are CD21^lo^, autoreactive and fail to be activated or proliferate after BCR stimulation[45]. However, we did not observe a correlation of CD21 MFI on naïve B cells and proportion of mature activated memory B cells (data not shown), making it difficult to conclude that mature activated B cells arise from naïve B cells in this setting.

We also observed that untreated HCV RF+ donors had increased viral levels in their serum in comparison to HCV RF- donors, consistent with Racanelli et al.[46] This raises questions about whether there is a direct contribution of virus on B cell subset distribution and activation. The advent of new DAA therapies offers the opportunity to evaluate the effects of rapid removal of HCV from the system without use of immune modulatory medications (IFN). Data here indicate a modest trend towards RF decline within the first 8 weeks of HCV IFN free DAA therapy. At the same time there was no significant reduction in the proportion of mature activated B cell proportions, activation state (CD86 expression) or cell cycle state (Ki67 expression) of HCV RF+ donors at week 8. Nonetheless, in HCV RF- donors the proportion of mature activated memory B cells normalized after 8 weeks of therapy to levels similar to those of uninfected donors. Therefore, RF and not viral load appears to be closely associated with B cell subset alterations in proportion and activation. Further investigation of the kinetics of normalization of RF using IFN free therapy should lend the best insight in B cell restoration. This information may help inform clinical management of the HCV RF+ infected patient during or shortly after DAA therapy.

## Supporting Information

S1 Fig Plasmablasts show enhanced CD86 and F(ab’)2 IgG promotes upregulation of pSYK Y348 in mature activated memory B cells of HCV RF+ donors. **(**A), Summary data of CD86 (%) in plasmablast (CD19+CD20- CD38+) in whole blood for uninfected donors (**▄ n = 20)**, HCV RF- **(● n = 17)**, and HCV RF+ donors (**▼, n = 17)**.(B) Phosflow assay on sorted mature activated memory B cells (3,000) that were stimulated with medium (shaded histogram), 5 minutes with 10μg of F (ab’)_2_ IgM Fc5_μ_ (dashed line) or 5 minutes with 10μg of F (ab’)_2_ IgG (solid line) of uninfected donors (n = 3) and HCV RF+ donors (n = 3).
(TIF)

S2 Fig Plasmablasts have high expression ki67 and naïve B cells of HCV RF+ donors fail to increase CD21. (A) Summary data of ki67 (%) in plasmablast (CD19+CD20- CD38+) in whole blood for uninfected donors (**▄ n = 20)**, HCV RF- **(● n = 17)**, and HCV RF+ donors (**▼, n = 17)**. (B)Summary data of Median Fluorescence Intensity of CD21 on naïve (CD19^+^CD20^+^CD21^+^CD27^-^) B cells. Kruskal- Wallis was used to analyzed differences among the three groups (*) of donors and Mann-Whitney U test (p≤0.05) was used between two groups of donors.
(TIF)

S1 Table IFN free direct acting antiviral study donor clinical characteristics. (DOCX)

## Abbreviations

ALT: alanine aminotransferase
AST: aspartate aminotransferase
APRI: AST/PLT ratio index
BCR: B cell receptor
CD: Cluster of differentiation
CFSE: Carboxyfluorescein succinimidyl ester
DAA: Direct acting antiviral therapy
ELISA: Enzyme Linked Immunosorbent Assay
FCS: fetal calf serum
HCV: hepatitis C virus
MFI: Median Fluorescence Intensity
PBMC: Peripheral blood mononuclear cells
PBS: phosphate buffered saline
PLT: Platelet
RF: rheumatoid factor
TLR: toll like receptor
UD: uninfected donors
