# Supplementary material for: Presence of Rheumatoid Factor during Chronic HCV Infection Is Associated with Expansion of Mature Activated Memory B-Cells that Are Hypo-Responsive to B-Cell Receptor Stimulation and Persist during the Early Stage of IFN Free Therapy
Source: PLoS One. 2015 Dec 9;10(12):e0144629. doi: 10.1371/journal.pone.0144629 (PMC4674123; doi:10.1371/journal.pone.0144629)
Supplement: S1 Table — (DOCX) [file pone.0144629.s003.docx]

**S1Table**. **IFN free direct acting antiviral study donor clinical characteristics**

| Variable | **Uninfected Donors** | **HCV Rheumatoid Factor negative (baseline)** | **HCV Rheumatoid Factor negative (week 8)** | **HCV Rheumatoid Factor positive (baseline)** | **HCV Rheumatoid Factor positive (week 8)** |
| --- | --- | --- | --- | --- | --- |
| **Number** | N= 10 | N=10 | N=10 | N=10 | N=10 |
| **Age,years** |  | 63.5 (31-75) |  | 60 (52-70) |  |
| **Plasma HCV RNA level, IU/mL** |  | 1,214,482  (317,877-11,391,000) | Not detected  (Less than 15 IU/mL) | 1,451,510 (126,800- 9,458,830) | Undetectable  (Less than 15 IU/mL |
| **Serum Albumin level, g/dL** | 4.1 (3.6-5.1) | 4 (3-4.30)^a^ | 3.65 (3.2-4.3) | 3.8 (2.3-4.20)^a^ | 3.8 (2.9-4.8) |
| **Platelet count, x10^3^/uL** | 249 (160-336) | 216 (69-400) | 217.5 (73-467)^c^ | 154 (57-329)^a^ | 187.5  (56-329)^b^ |
| **Serum AST level, U/L** | 23.5 (20-38) | 57 (27-106)^a^ | 22 (14-65)^b^ | 80 (26-211)^a^ | 30.5 (17-65)^b^ |
| **Serum ALT level, U/L** | 22.5 (16-41) | 54.5 (27-146)^a^ | 23.5 (11-39)^b^ | 79 (36-198)^a^ | 32.5 (18-77)^b^ |
| **APRI, (AST/PLT index)** | 0.233(0.134-0.351) | 0.791 (0.155-1.71)^a^ | 0.23 (0.070-0.730) | 1.33 (0.168-2.66)^a^ | 0.280 (0.110-1.19) |
| <0.4 | 9 (90%) | 4 (40%) | 9 (90%) | 2 (20%) | 6 (60%) |
| 0.4–1.5 | 1 (10%) | 4 (40%) | 1 (10%) | 3 (30%) | 4 (40%) |
| >1.5 |  | 2 (20%) |  | 5 (50%) |  |
| **HCV genotype %** | | | | | |
| 1 |  | 10 (100%) |  | 8 (80%) |  |
| 2/3 |  |  |  | 2 (20%) |  |
| **Duration of HCV**  **Infection** | | | | | |
| 1-10 years |  | 1 (10%) |  |  |  |
| >10 years |  | 8 (80%) |  | 9 (90%) |  |
| Unknown |  | 1 (10%) |  | 1 (10%) |  |
| **Sex** | | | | | |
| Male | 10 (100%) | 10 (100%) |  | 10 (100%) |  |
| Female |  |  |  |  |  |
| Ethnicity | | | | | |
| Caucasian | 3 (30%) | 3 (30%) |  | 5 (50%) |  |
| African American | 5 (50%) | 7 (70%) |  | 5 (50%) |  |
| Pacific Islander | 1 (10%) |  |  |  |  |
| Unknown | 1 (10%) |  |  |  |  |
| **RF level IU/mL** |  |  |  | 60% RF<100 IU/mL  40% RF >100 IU/mL | 60% RF<100 IU/mL  40% RF >100 IU/mL |

Values are expressed as median (range) for age, HCV RNA level, albumin, platelet, AST level, ALT level, APRI score, calculated as described previously [^42^](#_ENREF_42), and absolute CD19+ count. Numbers and proportions of subjects within each category are given for HCV genotype, duration of HCV infection, sex, ethnicity and RF. Abbreviations: ALT, alanine aminotransferase; APRI, AST-to-Platelet ratio index; AST, aspartate aminotransferase; HCV, hepatitis C virus, and RF, rheumatoid factor.  ^a^ p< .05 compared with uninfected donors; ^b^ p< .05 compared between baseline and w8.
